# Behavioral Disturbances in a Patient with ADHD and Borderline Intellectual Functioning: A Clinical and Therapeutic Challenge

**DOI:** 10.1192/j.eurpsy.2026.11099

**Published:** 2026-07-31

**Authors:** C. P. C. Alonso, J. G. Ruiz, E. L. Bardon, M. M. Muñoz, I. G. Rodriguez, P. F. Perea

**Affiliations:** 1 Psiquiatria, Sacyl, Leon; 2 psiquiatria, Sacyl, León, Spain

## Abstract

**Introduction:**

Behavioral disturbances in patients with Attention-Deficit/Hyperactivity Disorder (ADHD) and borderline intellectual functioning represent a significant clinical challenge. The presence of anxiety symptoms, disorganized thought patterns, poor functional autonomy, and limited insight complicates therapeutic approaches. Comorbidity with agoraphobia, paranoid ideation, and distrust toward support figures, combined with poor social support, can lead to prolonged hospitalizations and the need for structured rehabilitation.

**Objectives:**

To present the case of a patient diagnosed with ADHD, agoraphobia, and borderline functioning, with progressive functional deterioration, behavioral alterations, and social isolation, requiring involuntary psychiatric hospitalization and comprehensive evaluation for referral to rehabilitation services.

**Methods:**

We describe the clinical follow-up during her admission to a psychiatric inpatient unit, including structured interviews with the patient and family, additional tests (EEG, psychological evaluation), daily clinical observations by the multidisciplinary team, and pharmacological adjustments. The case presentation details the initial symptoms, response to hospitalization, clinical and functional evolution, and the rationale for referral to a rehabilitation unit.

**Results:**

We present the case of a 39-year-old woman with a history of ADHD, agoraphobia, borderline IQ, progressive isolation, and growing distrust towards her family and healthcare providers. She presented with marked anxiety, emotional lability, disorganized speech, suspicion, and poor insight. Upon admission, she was highly demanding, psychomotor agitated, repetitive in speech, and showed impaired reality testing, though without suicidal or aggressive ideation.

During hospitalization, under pharmacological treatment and environmental containment, she gradually improved: anxiety levels decreased, tolerance to frustration increased, speech became more coherent and goal-directed, and she began to accept both the admission and the need for help. Various pharmacological adjustments were implemented, notably the introduction of paroxetine and aripiprazole, progressive reduction of benzodiazepines, and as-needed antipsychotic medication for agitation. The patient accepted referral to a rehabilitation unit to continue structured intervention and improve her functionality.

**Conclusions:**

This case highlights the challenges of managing patients with complex psychiatric conditions, poor insight, and significant social vulnerability. A multidisciplinary approach and structured environment enabled clinical stabilization and the beginning of awareness and recovery. Rehabilitation referral was essential for promoting autonomy and long-term prognosis.

**Disclosure of Interest:**

None Declared

